# TIMP2 facilitates CIRI through activating NLRP3-mediated pyroptosis

**DOI:** 10.18632/aging.204696

**Published:** 2023-05-12

**Authors:** Shaoyong Shi, Chongyang Zhang, Jiaxiang Liu

**Affiliations:** 1 Department of Prehospital Emergency Care, Qinhuangdao First Hospital, Qinhuangdao 066000, China

**Keywords:** TIMP2, ERK1/2, NLRP3, pyroptosis, cerebral ischemia reperfusion, seahorse organization

## Abstract

This study aimed to investigate the underlying mechanisms of cerebral ischemia-reperfusion injury (CIRI) in mice using CIR and hypoxia/reoxygenation (H/R) cell models. The study evaluated brain tissue weight, pathological injury, and changes in the expression levels of TIMP2, p-ERK1/2 and NLRP3-mediated pyroptosis-related proteins in brain tissues and hippocampal neurons of CIR mice using established methods such as dry/wet weight measurement, HE staining, qPCR, TUNEL assay, and Western blotting. The results demonstrated a significant increase in brain water content and neuronal apoptosis rate in the experimental groups compared with those in the control group. In particular, the I/R+TIMP2 group showed the highest increase. Additionally, the control group exhibited a clear brain tissue structure, neatly and densely arranged cells with normal morphology, and evenly stained and clear hippocampal tissues. However, the I/R group showed hippocampal structure disorders, interstitial edema, deep nuclear staining, karyopyknosis, and karyorrhexis in brain tissues. The study results further revealed that TIMP2 could aggravate the pathological damage of brain tissues in the I/R+TIMP2 group compared with the I/R group and significantly reduced it in the TIMP2-KD group. Furthermore, the Western blotting results demonstrated that the protein expression levels of TIMP2, p-ERK1/2, t-ERK1/2, NLRP3, IL-1β, IL-18, GSDMD, Caspase-1, and ASC in brain tissues and hippocampal neurons were significantly higher in the experimental groups than those in the control group. The I/R+TIMP2 group displaying the highest increase and the TIMP2-KD group showing a significant decrease. In conclusion, TIMP2 can contribute to the occurrence and progression of CIRI by activating NLRP3-mediated pyroptosis.

## INTRODUCTION

Cerebrovascular disease is a prevalent health condition that poses a significant threat to the well-being of older individuals. This condition is characterized by high morbidity, disability, and mortality rates [1, 2]. Cerebral ischemia-reperfusion (I/R) injury (CIRI) is a condition that results in severe cerebral injury and related dysfunction due to blood reperfusion in the ischemic area [3, 4].

Nod-like receptor protein 3 (NLRP3) inflammasomes have emerged as essential players in the pathogenesis of central nervous system diseases. In the case of stroke, multiple types of inflammasomes, including NLRP1, NLRP3, NLRC4, and AIM2 inflammasomes, are activated and expressed [5]. Among them, NLRP3 inflammasomes have been the subject of extensive research. Literature reports that NLRP3 is present in microglia, astrocytes, endothelial cells [5, 6], and even neurons in brain diseases [6, 7]. In ischemic stroke, NLRP3 inflammasomes contribute to the progression of cerebral injury. Cerebral ischemia triggers a cascade of reactions, including the production of endogenous reactive oxygen species (ROS), inflammatory responses, and autophagy due to mitochondrial dysfunction. Under various stimuli, such as the production of endogenous ROS and the release of cathepsin B by lysosomes [7], NLRP3 associates with ASC and pro-Caspase-1 to form inflammasomes, leading to the secretion of interleukin-1β (IL-1β) and IL-18. This process generates activated Caspase-1 through cleavage and promotes secretion, leading to tissue damage in conjunction with other injury pathways [8]. Hence, the NLRP3 inflammasome signaling pathway may serve as a critical mediator of inflammatory and immune responses.

Tissue inhibitor of metalloproteinase 2 (TIMP2) is a crucial endogenous inhibitor of matrix metalloproteinase-2 (MMP-2) [9], and its expression changes are associated with the development of ischemic stroke and intracerebral hemorrhage [10]. The overexpression of TIMP2 has been observed to stimulate the expressions of extracellular signal-regulated kinase 1/2 (ERK1/2) and c-Jun N-terminal kinase 1/2 (JNK1/2) in the mitogen-activated protein kinase (MAPK) pathway [11]. ERK1/2, the first identified MAPK signaling pathway, is known to be activated by oxidative stress through upstream factors such as calcium channels, receptor tyrosine kinases, RAS, and Src [12]. ERK1/2 signaling plays a vital role in the regulation of various physiological processes, including cell proliferation, differentiation, and survival and death [13], and is regarded as an important event in NLRP3 inflammasome activation [8, 14].

In this study, therefore, a CIR mouse model and a hypoxia/reoxygenation (H/R) cell model were established to explore the underlying molecular mechanism involved in the development of CIRI in mice through *in vitro* and *in vivo* experiments.

## MATERIALS AND METHODS

### Animal modeling and grouping

Male C57 mice (6–8 weeks old, 25 g) were purchased from the Laboratory Animal Center of Hebei Medical University, and all animal experiments adhered to the guidelines set by the Animal Ethics Committee. The mice were acclimatized to standard laboratory conditions for one week before experiments and were randomly divided into one of the four groups: control group, I/R group, I/R+TIMP2 group, and TIMP2-KD group. The mice were fixed in a supine position and administered an intraperitoneal injection of sodium pentobarbital for anesthesia. The neck skin was disinfected, and an incision was made along the cervical midline to expose the right common carotid artery, external carotid artery, and internal carotid artery, which were then bluntly separated. The common carotid artery and external carotid artery were ligated, and a suture was inserted through a small incision in the common carotid artery to the common carotid artery. Finally, the skin was sutured, and after 90 min of ischemia followed by 24 h of reperfusion, the suture was removed.

### Cell culture and treatment

Hippocampal neurons were harvested from neonatal mice to establish the H/R cell model for *in vitro* experiments. These neurons were cultured routinely in RPMI1640 medium supplemented with 10% FBS and 100 U/L of penicillin/streptomycin in a 5% CO_2_ incubator at 37°C. Upon confluence in the culture flask, the cells were trypsinized using 0.25% trypsin, cultured in a single cell suspension, and maintained in RPMI1640 medium with regular medium changes. Subsequently, 2 × 10^5^ single-cell suspensions of logarithmically growing cells were inoculated into 6-well plates and cultured with a serum-free medium in an incubator (5% CO_2_, 1% O_2_, and 94% N_2_) for 3 h. The cells were then cultured in a serum-containing medium with 5% CO_2_ at 37°C for 3 h for reoxygenation. Thereafter, the cells were divided into a control group, a I/R group, a I/R+TIMP2 group, and a TIMP2-KD group for cell experiments. In the I/R+TIMP2 group, cells required the transfection of circ-TIMP2 overexpression plasmids. To construct a circ-TIMP2 overexpression vector, a pre- and post-circular frame of TIMP2 was generated and added to the pLCDH-ciR vector (Gene Seed Biotechnology Ltd.) for transcript cycling. The anteroposterior circular frame consisted of endogenous flanking genome sequences with EcoRI and BamHI restriction sites, respectively. The 3,122 bp target sequence included the EcoRI site, shear acceptor AG, circ-TIMP2 sequence, splicing donor GT, and BamHI site. The PCR product was cloned between two frames, and circRNA was amplified using specific divergent primers for circ-TIMP2 back-splicing ligation. In the TIMP2-KD group, cells were transfected with gRNA plasmids and donor fragments using the TIMP-2 Human Knockout Kit (CRISPR, KN209796, Origen Rockville, Maryland, USA). Two gRNA plasmids targeting the TIMP-2 sequence were used: KN209796G1, TIMP-2 gRNA vector 1,3-5 μg in the pCas-Guide vector, with target sequence: AGCAGCTGCAGGCGTCGGCC (gRNA1), and KN209796G2, TIMP-2 gRNA vector in pCas-Guide vector 2, 3–5 μg, with target sequence: CGCACCCTGCGGCTGGCGGCT (gRNA2). The linear donor fragment KN409796D contained green fluorescent protein (GFP) and puromycin (LoxP-EF1A-tGFP-P2A-Puro-LoxP). Cell transfection was performed using one gRNA/Cas-9 vector and donor fragments (GFP and puromycin) plus Lipofectamine 2000 at a 1:3 ratio.

### Dry/wet weight measurement

Following the sacrifice of the mice by cervical dislocation, their brains were carefully harvested, with the olfactory bulb, cerebellum, and lower brain stem removed. The wet weight of the brains was then measured. Subsequently, the brains were placed in a petri dish, and the surface was dried with filter paper to remove excess moisture. The brains were then oven-dried at 90°C until a constant weight was achieved, representing the dry weight. The brain water content and cerebral index were calculated using the formula: brain water content (%) = (wet weight – dry weight)/wet weight × 100%.

### Hematoxylin-eosin (HE) staining

The brain tissues were initially fixed using 10% formaldehyde for 48 h, followed by dehydration in ethanol, transparency in xylene, embedding in paraffin, and sectioning into 5 μm-thick slices. Subsequently, the sections were stained with HE and mounted using neutral balsam. Finally, brain tissue injury was evaluated by visual inspection under a fluorescence microscope (400×).

### Quantitative polymerase chain reaction (qPCR)

The TRIzol method was employed to extract total RNA from brain tissues. The synthesized cDNA was obtained using the BeyoRT^™^ II cDNA First Strand Synthesis Kit, followed by qPCR experiments with SYBR Green. The qPCR reaction conditions included pre-denaturation at 95°C for 5 min, followed by 40 cycles of denaturation at 95°C for 30 s, annealing at 65°C for 45 s, and extension at 72°C for 5 min. The forward primer for TIMP2 was 5′-AAGAACATCAACGGGCACCA-3′, and the reverse primer was 5′-TGGACCAGTCGAAACCCTTG-3′.

### Detection of neuronal apoptosis by TUNEL assay

Apoptosis was assessed using TUNEL kits according to the manufacturer’s instructions. Briefly, TUNEL-positive cells with brown-stained nuclei were observed under a microscope, while normal nuclei were stained blue. Apoptotic cells were counted using ImageJ software, with five randomly-selected fields examined under a fluorescence microscope. The apoptosis index (AI) was determined by the formula: AI = the number of positive cells in each field/total cells in each field × 100%.

### Western blotting

The total protein was extracted from hippocampal tissues and cells using BCA kits, and its concentration was measured. An equal number of protein samples were denatured at 100°C for 5 min and separated by SDS-PAGE. These samples were then transferred onto a PVDF membrane and incubated with primary antibodies against TIMP2 (ab180630), phosphorylated extracellular signal-regulated kinase 1/2 (p-ERK1/2) (ab278538), NLRP3 (ab263899), IL-1β (ab216995), IL-18 (ab207323), gasdermin D (GSDMD) (ab209845), cysteine-dependent aspartate-directed protease-1 (Caspase-1) (ab207802), apoptosis-associated speck-like protein containing a CARD (ASC) (ab155970), and GAPDH (ab2293) overnight. The membrane was washed and then incubated again with horseradish peroxidase-labeled secondary antibodies at 4°C for 2 h. Finally, the protein samples were treated with ECL solution and exposed to detect the gray value using ImageJ software.

### Statistical analysis

SPSS19.0 software was used for statistical analysis, and the data were expressed as mean ± standard deviation. Independent *t*-tests were used for pairwise comparison and one-way analysis of variance for the difference between groups. *P* < 0.05 was considered statistically significant.

## RESULTS

### Changes in brain water content

**Figure 1:**
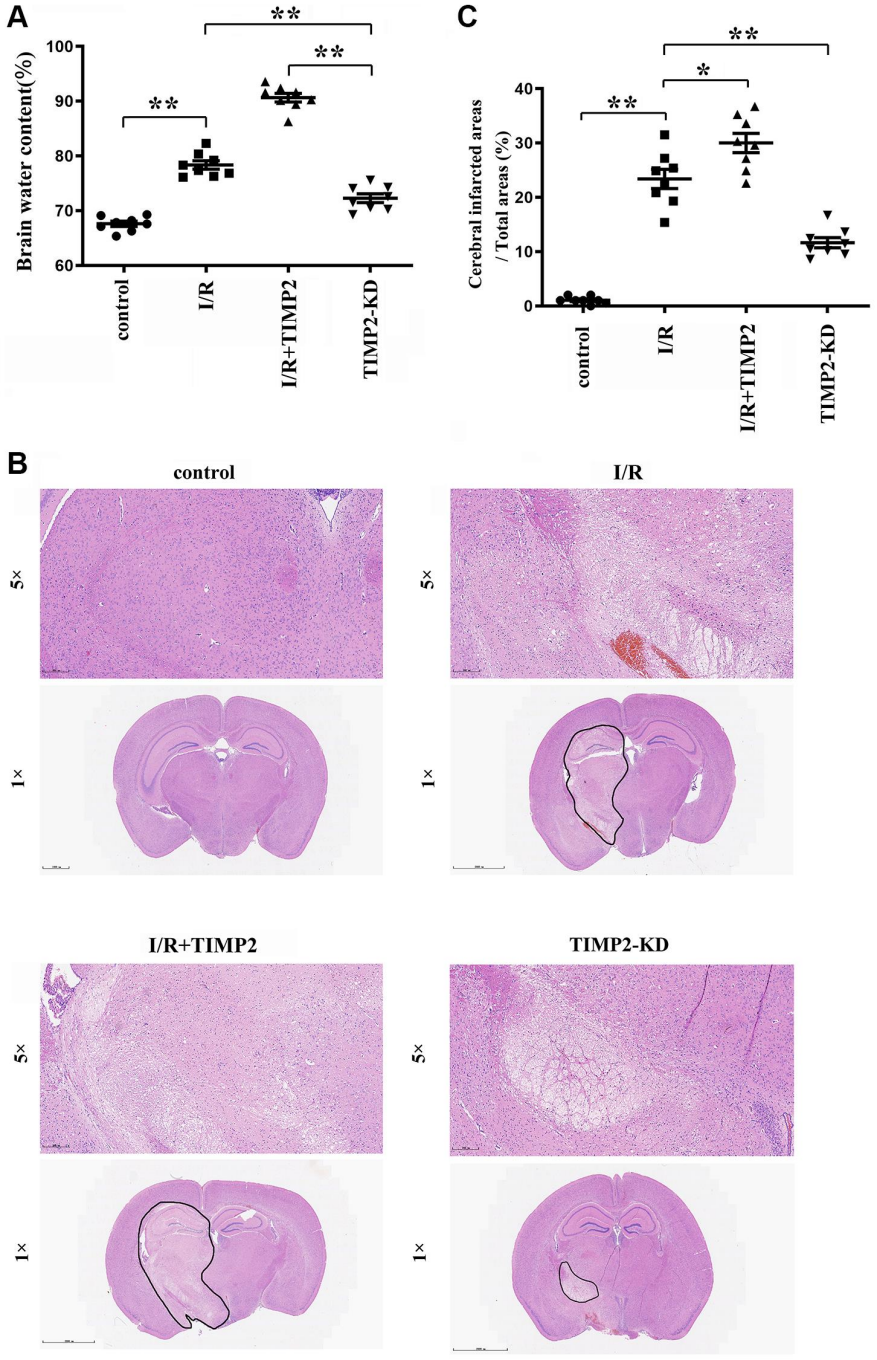
**TIMP2 can aggravate brain water content and brain tissue damage in CIRI mice.** (**A**) Brain water content is measured in the control group, I/R group, and I/R+TIMP2 group. Brain water content is significantly higher in the I/R group than that in the control group. Furthermore, the use of TIMP2 further increases brain water content in the I/R group. In contrast, brain water content is significantly reduced in the TIMP2-KD group. (**B**, **C**) Pathological changes in brain tissues are observed using HE staining in the control group, I/R group, and I/R+TIMP2 group. No pathological changes are observed in the control group, whereas mice in the I/R+TIMP2 group showed larger pathological changes compared with the I/R group. Conversely, the pathological area in the brain tissues of mice in the TIMP2-KD group becomes smaller. ^**^P < 0.01, ^*^P < 0.05.

The dry/wet weight measurement results revealed a noteworthy increase in brain water content in the experimental groups subjected to CIR compared with that in the control group, with a further increase observed in the I/R+TIMP2 group compared with that in the I/R group. Remarkably, the TIMP2-KD group displayed a significant decrease in brain water content compared with the I/R group, underscoring the exacerbating effect of TIMP2 on brain water content in this setting. These observations suggested that brain water content could serve as a valuable indicator of the severity of CIRI in mice (Figure 1A).

### Pathological injury degree of brain tissues

In the control group, brain tissues exhibited clear structures, with densely arranged cells showing normal morphology and evenly stained hippocampal tissues. In contrast, the I/R group showed hippocampal structure disorders, interstitial edema, deep nuclear staining, karyopyknosis, and karyorrhexis in brain tissues. Notably, the I/R+TIMP2 group exhibited a further worsening of the pathological injury of brain tissues compared with the I/R group. In addition, the TIMP2-KD group displayed a significant reduction in pathological injury compared with the I/R group. The findings of this study indicated that TIMP2 aggravates the pathological injury of the brain of mice during CIR (Figure 1B, 1C).

### TIMP2 was highly expressed in the brain tissues of CIRI mice

**Figure 2:**
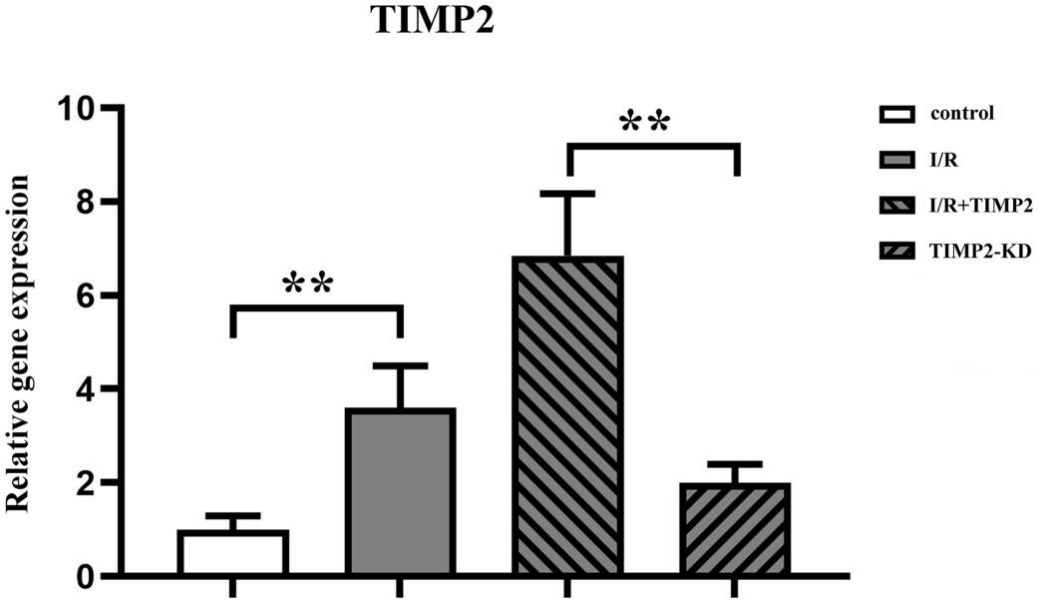
**TIMP2 aggravates brain tissue injury in CIRI mice.** QPCR is used to measure the expression of TIMP2 in the control, I/R, and I/R+TIMP2 groups. The expression of TIMP2 is significantly higher in the I/R group and the I/R+TIMP2 group than that in the control group. In addition, the expression of TIMP2 is significantly higher in the I/R+TIMP2 group than that in the I/R group. ^**^P < 0.01, ^*^P < 0.05.

The qPCR analysis results showed a significant upregulation of TIMP2 expression in both the I/R group and I/R+TIMP2 group compared with the control group, with a significantly high expression observed in the I/R+TIMP2 group. In contrast, the TIMP2-KD group exhibited a significant downregulation of TIMP2 expression relative to the I/R group. These above results demonstrated the elevated expression of TIMP2 in the brain tissues of mice during CIR and successful modeling across all experimental groups (Figure 2).

### Neuronal apoptosis in CIRI mice

**Figure 3:**
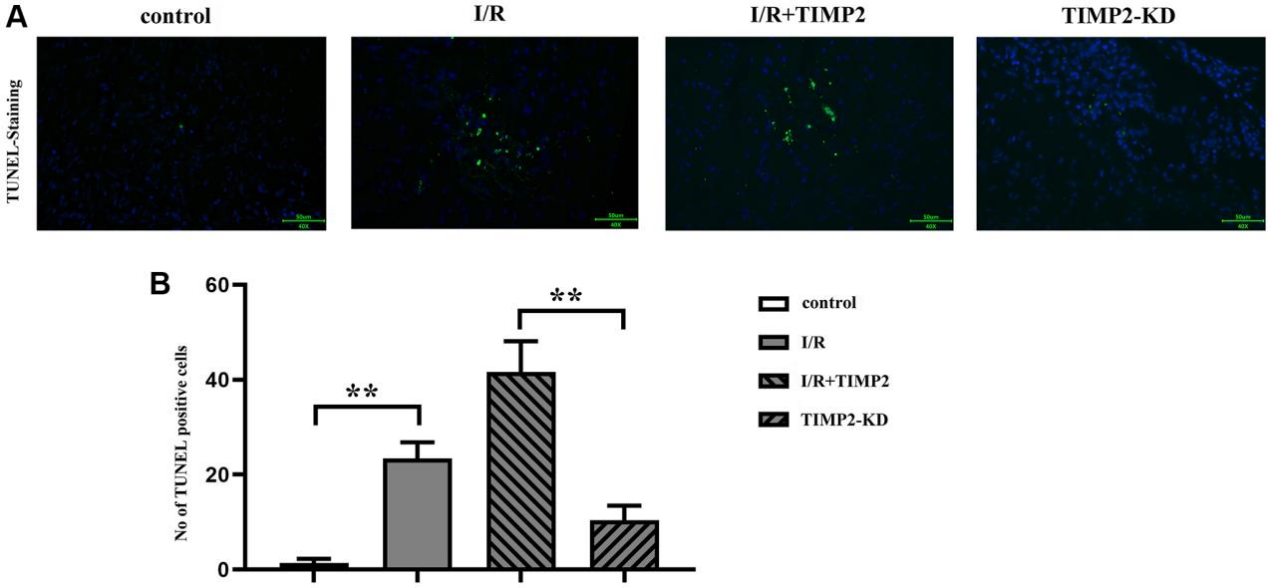
**TIMP2 can aggravate neuronal apoptosis in CIRI mice.** (**A**, **B**) TUNEL staining is performed to observe neuronal apoptosis in the control, I/R, and I/R+TIMP2 groups. In the control group, there is basically no green fluorescence, while the number of TUNEL-positive cells is larger in the I/R and I/R+TIMP2 groups compared with that in the control group. Furthermore, the number of TUNEL-positive cells is significantly larger in the I/R+TIMP2 group than that in the I/R group. In contrast, the number of TUNEL-positive cells is significantly smaller in the TIMP2-KD group than that in the I/R group. These results suggested that TIMP2 may aggravate neuronal apoptosis in mice with CIRI. ^**^P < 0.01, ^*^P < 0.05.

The results of TUNEL staining showed a significant increase in the number of TUNEL-positive cells in the I/R and I/R+TIMP2 groups compared with that in the control group. Moreover, the number of TUNEL-positive cells in the I/R+TIMP2 group was further increased compared with the I/R group, indicating a higher apoptosis rate of neuronal cells in this group. In contrast, the number of apoptotic cells in the TIMP2-KD group was significantly decreased relative to that in the I/R group. The above findings suggested that TIMP2 promotes neuronal apoptosis and exacerbates brain damage in mice with CIRI (Figure 3).

### TIMP2 enhanced the occurrence and development of CIRI by activating NLRP3-mediated pyroptosis

**Figure 4:**
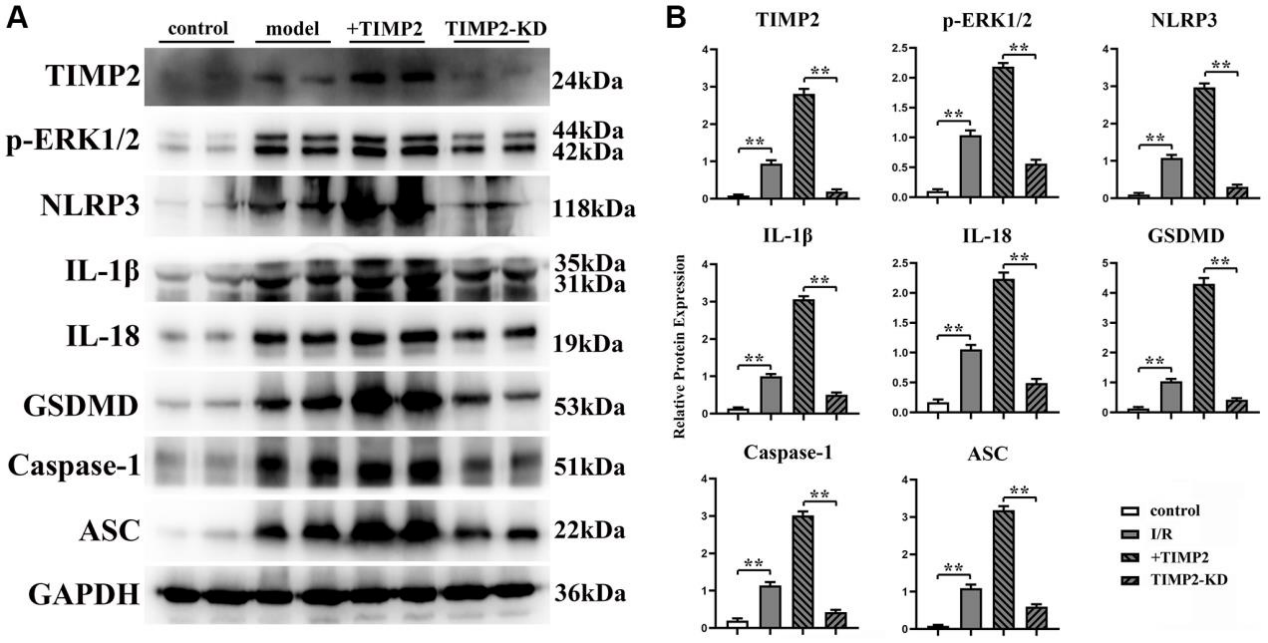
**TIMP2 can stimulate NLRP3-mediated cell apoptosis in mice with CIRI.** (**A**) Western blotting is performed to detect the expression levels of TIMP2, p-ERK1/2, NLRP3, and IL-1β in each group. The protein banding diagram of IL-18, GSDMD, Caspase-1, and ASC; (**B**) The protein expressions of TIMP2, p-ERK1/2, NLRP3, IL-1, IL-18, GSDMD, Caspase-1, and ASC in the control, I/R, I/R+TIMP2, and TIMP2-KD groups. It can be observed that TIMP2 can upregulate the expression of these proteins, indicating that TIMP2 can aggravate cell pyroptosis in mice with CIRI. ^**^P < 0.01.

**Figure 5:**
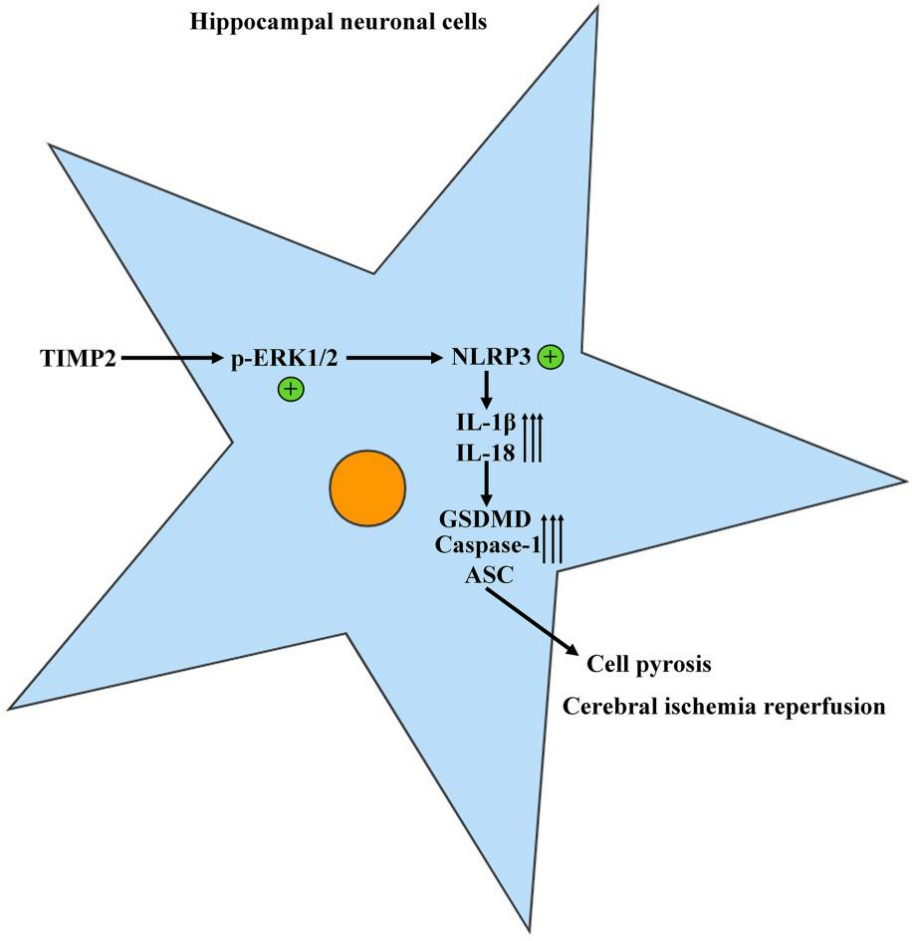
TIMP2 can promote the progression of CIRI by activating the NLRP3-mediated cell pyroptosis.

The protein expression levels of TIMP2, p-ERK1/2, t-ERK1/2, NLRP3, IL-1β, IL-18, GSDMD, Caspase-1, and ASC in brain tissues and hippocampal neurons were evaluated using Western blotting. The results revealed that the expression levels of these proteins were significantly higher in the experimental groups than those in the control group. Moreover, the expression levels were further increased in the I/R+TIMP2 group compared with those in the I/R group. Conversely, the expression levels of the above-mentioned proteins were significantly lower in the TIMP2-KD group than those in the I/R group (Figure 4). In summary, TIMP2 was found to facilitate the occurrence and progression of CIRI by activating NLRP3-mediated pyroptosis. The findings indicated that TIMP2 is highly expressed in CIR, promoting the pyroptosis of hippocampal somatic cells through the NLRP3 signaling pathway-related proteins, ultimately aggravating CIRI (Figure 5).

## DISCUSSION

The etiology of cerebral ischemia-induced brain tissue injury is multifactorial, involving inflammation, oxidative stress, apoptosis, energy metabolism, and calcium overload [15–17]. The reperfusion of blood in the ischemic region leads to severe cerebral injury and related dysfunctions, especially neuronal and neuroglia damage. To understand the underlying mechanisms of CIRI, this study aimed to establish CIR mouse and H/R cell models to explore the mechanism underlying the occurrence and development of CIRI in mice through *in vitro* and *in vivo* experiments.

Research has established a correlation between inflammasome expression and various brain disorders, including Alzheimer’s disease, Parkinson’s disease, and stroke [18, 19]. Recent studies have confirmed the close association between NLRP3 inflammasomes and CIRI [19, 20]. NLRP3 inflammasomes are reliable markers for detecting post-cerebral infarction cell injury and key mediators for inflammatory response regulation. Activated NLRP3 inflammasomes have been found to enhance the release of inflammatory factors and contribute to pathological inflammatory responses in CIR [21]. NLRP3 is a multimeric protein complex composed of NLR family proteins, ASC, and Caspase-1 precursor [22]. This study revealed significantly increased protein expression levels of NLRP3, IL-1β, IL-18, GSDMD, Caspase-1, and ASC in brain tissues and hippocampal neurons in the experimental groups compared with those in the control group, suggesting that activated NLRP3 inflammasomes are involved in CIRI in mice. The above findings are consistent with those reported by Ma et al. [8], which demonstrated upregulated protein expressions of NLRP3, ASC, Caspase-1, and IL-1β after CIRI in rats. Ma et al. also observed pronounced hemodynamic changes in the cerebral ischemic and infarction areas, compromised blood-brain barrier permeability, increased volume of hydrocephalus and number of apoptotic cells, and deteriorated neurological function.

This study revealed that CIR significantly upregulated the protein expression of TIMP2 in brain tissues, suggesting that TIMP2 is highly expressed in the brain tissues of CIRI mice. Bo Liang et al. reported that TIMP2 knockdown promoted cell proliferation and inhibited apoptosis [23]. This is consistent with the current study’s finding that the neuronal apoptosis rate significantly rose in the experimental groups compared with that in the control group and further rose in the I/R+TIMP2 group compared with that in the I/R group. Conversely, the TIMP2-KD group had a significantly lower neuronal apoptosis rate than the I/R group. Western blotting analysis revealed that the expressions of TIMP2 and p-ERK1/2 in brain tissues and hippocampal neurons significantly rose in the I/R+TIMP2 group compared with those in the I/R group, while the TIMP2-KD group had significantly lower protein expressions than the I/R group, indicating that the ERK pathway is activated after CIR. ERK1/2 was the first MAPK signaling pathway discovered, and ERK1/2 signals play a vital role in controlling cell proliferation and differentiation, regulating key physiological processes such as cell survival and death [13]. ERK1/2 signal transduction is a key event in the activation of NLRP3 inflammasomes [8, 14]. The expression levels of pyroptosis-related proteins mediated by NLRP3 were in line with TIMP2 and p-ERK1/2, indicating a further increase in the I/R+TIMP2 group compared with the I/R group. In contrast, the expression levels of these proteins were significantly lower in the TIMP2-KD group than in the I/R group, providing evidence that TIMP2 can activate the NLRP3-mediated pyroptosis. The control group showed normal brain tissue structure, with neatly arranged cells, normal morphology, and evenly stained and clear hippocampal tissues. Additionally, the pathological injury of brain tissues was more severe in the I/R+TIMP2 group than that in the I/R group. In contrast, pathological damage to brain tissues was reduced in the TIMP2-KD group. It can be inferred that TIMP2 plays a crucial role in the occurrence and progression of CIRI in mice by activating NLRP3-mediated pyroptosis.
